# The Relationship between Intelligence and Divergent Thinking—A Meta-Analytic Update

**DOI:** 10.3390/jintelligence9020023

**Published:** 2021-04-20

**Authors:** Anne Gerwig, Kirill Miroshnik, Boris Forthmann, Mathias Benedek, Maciej Karwowski, Heinz Holling

**Affiliations:** 1 Institute of Psychology, University of Münster, 48149 Münster, Germany; anne.gerwig@web.de (A.G.); holling@uni-muenster.de (H.H.); 2 Faculty of Psychology, Saint Petersburg State University, 199034 Saint Petersburg, Russia; cyril.miroshnik@gmail.com; 3 Institute of Psychology in Education, University of Münster, 48149 Münster, Germany; 4 Institute of Psychology, University of Graz, 8010 Graz, Austria; mathias.benedek@uni-graz.at; 5 Institute of Psychology, University of Wroclaw, 50-527 Wroclaw, Poland; maciej.karwowski@uwr.edu.pl

**Keywords:** divergent thinking, intelligence, fluid intelligence, crystallized intelligence, meta-analysis

## Abstract

This paper provides a meta-analytic update on the relationship between intelligence and divergent thinking (DT), as research on this topic has increased, and methods have diversified since Kim’s meta-analysis in 2005. A three-level meta-analysis was used to analyze 849 correlation coefficients from 112 studies with an overall *N* = 34,610. The overall effect showed a significant positive correlation of *r* = .25. This increase of the correlation as compared to Kim’s prior meta-analytic findings could be attributed to the correction of attenuation because a difference between effect sizes prior-Kim vs. post-Kim was non-significant. Different moderators such as scoring methods, instructional settings, intelligence facets, and task modality were tested together with theoretically relevant interactions between some of these factors. These moderation analyses showed that the intelligence–DT relationship can be higher (up to *r* = .31–.37) when employing test-like assessments coupled with be-creative instructions, and considering DT originality scores. The facet of intelligence (g vs. g_f_ vs. g_c_) did not affect the correlation between intelligence and DT. Furthermore, we found two significant sample characteristics: (a) average sample age was positively associated with the intelligence–DT correlation, and (b) the intelligence–DT correlation decreased for samples with increasing percentages of females in the samples. Finally, inter-moderator correlations were checked to take potential confounding into account, and also publication bias was assessed. This meta-analysis provides a comprehensive picture of current research and possible research gaps. Theoretical implications, as well as recommendations for future research, are discussed.

## 1. Introduction

All human discoveries and inventions are marked by intelligence and creativity. A better understanding of their association will not only update psychological theories but also improve educational practices. Decades of intensive inquiry have resulted in the accumulation of diverse theoretical perspectives and contradicting empirical findings on how intelligence and creativity are related (Plucker and Esping 2015; Plucker et al. 2020; Silvia 2015; Sternberg and O’Hara 1999). Even though both constructs are multi-faceted, the hottest part of the debate—both theoretically and historically—focuses on the association between psychometric intelligence and divergent thinking ability (DT; Plucker and Renzulli 1999; Plucker et al. 2020; but see also Xu et al. 2019). DT is a crucial component of cognitive creative potential (Runco and Acar 2012, 2019), albeit it is not synonymous with creativity (Parkhurst 1999; Runco 2008). Indeed, the relationship between intelligence and DT has been discussed in creativity research for decades (Plucker and Esping 2015; Plucker et al. 2020; Silvia 2015; Sternberg and O’Hara 1999). Even though early research understood DT and intelligence as distinct concepts (Getzels and Jackson 1962; Wallach and Kogan 1965), the question remains whether there are similarities between the constructs or common cognitive processes behind them (Plucker and Esping 2015). Fifteen years ago, Kim (2005) conducted a meta-analysis and found a positive but relatively small correlation between intelligence and DT of *r* = .174 (95% CI: .165, .183), indicating that the two constructs share only 3% of the variance. However, several limitations have been discussed in Kim’s (2005) work, and some researchers have criticized that a subset of the included studies was too old to reflect current theories of intelligence (Plucker and Esping 2015). Moreover, since this meta-analysis, renewed interest in DT measurement issues led to advancements with respect to scoring approaches, statistical methods, and theoretical developments (Runco and Acar 2019; Reiter-Palmon et al. 2019; Silvia 2015). A lot of effort has been taken by researchers considering different aspects such as instructional settings or a more detailed conceptualization of intelligence that may moderate the link between intelligence and DT. Hence, it is time for a meta-analytic update to shed light onto the still not fully answered question about the relationship between intelligence and DT as a vital facet of creative thinking.

## 2. The Relationship between Intelligence and DT

DT describes the process of generating a variety of solutions (Guilford 1967). In the context of the standard definition of creativity, responses are considered to be creative if they are novel/original and appropriate/effective (Runco and Jaeger 2012). Following this reasoning, the ability to come up with various ideas (evaluated as being creative) constitutes DT ability as an indicator of creative potential (Runco and Acar 2012). Flexible, critical, and playful thinking, as well as problem-solving ability, and the willingness to accept ambiguous situations are expected to facilitate DT (Karwowski et al. 2016a; Plucker and Esping 2015). In a recent review, Plucker and Esping (2015) outlined various points of view regarding the question of where to locate DT: as a facet of intelligence, as a result of intelligence, or as a separate construct, sharing cognitive abilities with intelligence. A fair number of intelligence researchers consider DT as a subcomponent of intelligence (Carroll 1997; Guilford 1967; Jäger 1982; Karwowski et al. 2016a). (Carroll 1997; see also Chrysikou 2018; Dietrich 2015) stated that DT requires several mental abilities, such as the speed of retrieval (e.g., Forthmann et al. 2019), knowledge (e.g., Weisberg 2006), fluid intelligence (e.g., Beaty et al. 2014; Nusbaum et al. 2014), and motor skills (e.g., the ability to write quickly; see: Forthmann et al. 2017). This view emphasizes that multiple factors might moderate the strength of the association between intelligence and DT. Although DT and intelligence have been seen as somewhat different constructs in the past (Getzels and Jackson 1962; Wallach and Kogan 1965), some researchers have now adopted the view that the constructs might be more similar than previously thought (Silvia 2015). To conclude, a robust empirical examination of the relationship between intelligence and DT is expected to clarify the theoretical relationship of these constructs.

Creativity research has witnessed several methodological and conceptual developments over the past two decades. First of all, today latent variable analyses provide possibilities to separate the true variance of DT from error-variance resulting from the task- or procedure-specific factors and other unknown sources. As a result, effect sizes can be estimated more accurately (Silvia 2015). For example, Silvia (2008) reanalyzed the data of Wallach and Kogan’s (1965) study on the relationship between intelligence and DT with 151 children using a structural equation model. Compared to the negligible correlation of *r* = .09 reported by Wallach and Kogan (1965), Silvia (2008) found a more substantial relationship between the latent factor intelligence and the latent factor DT (β = 0.22), demonstrating that the observed correlations deflate the true relationship between the two constructs (Silvia 2015). Hence, increased use of appropriate corrections for unreliability—for example, employing structural equation modeling—forms one reason for the renaissance of the debate on the relationship between intelligence and DT.

Additionally, many researchers have focused on a bundle of different possible moderators of the relationship and investigated associations between these constructs’ specific facets. Some found evidence for the role of fluid intelligence (e.g., Beaty et al. 2014; Nusbaum and Silvia 2011), broad retrieval ability (e.g., Forthmann et al. 2019; Silvia et al. 2013), and crystallized intelligence (e.g., Cho et al. 2010) as factors that differentiated the intelligence–DT link. For example, Silvia (2015) emphasized that fluid intelligence plays a crucial role for DT. However, he and his colleagues used DT tests with explicit *be-creative* instruction, evaluated ideas using subjective ratings, and statistically controlled for measurement errors (e.g., Nusbaum and Silvia 2011; see more details on instructions and scoring methods in Section 3.3 below). Silvia (2015) concluded that contrary to the previous position that DT is closely linked to crystallized intelligence and hence depends on how much a person knows (Mednick 1962; Weisberg 2006), fluid intelligence (i.e., reasoning and processing of information) plays a more important role than expected and has to be taken into account. In this regard, it is especially interesting that building on a more comprehensive set of cognitive abilities, Weiss et al. (2020a) reported quite comparable correlations between DT and general intelligence (encompassing g_f_, g_c_, mental speed, and working memory) and between DT and crystallized intelligence. In this vein, it is further notable that measures of g_r_ (i.e., broad retrieval ability; Carroll 1993) such as verbal fluency tasks reflect a combination of knowledge (mapping onto g_c_) and strategic retrieval (mapping onto g_f_). In turn, a moderate to strong relationship between g_r_ and DT originality/creative quality was found (Forthmann et al. 2019; Silvia et al. 2013). However, it is unclear whether the relationship between intelligence and DT is moderated by type of intelligence (fluid vs. crystallized intelligence), instructions used for DT assessment, scoring procedures, and time on task (Forthmann et al. 2020a; Preckel et al. 2011). It further remains unclear if these moderators influence the relationship between DT and intelligence independently or if specific interactions of these factors can explain differences across studies. Taken together, creativity research provides mixed results so that the question of the interrelation between the constructs has not fully been answered yet (Batey and Furnham 2006; Silvia 2015).

Recent works pay closer attention to more processual and cognitive mechanisms standing behind intelligence–DT links. It has been demonstrated that cognitive control (and executive functions more broadly) are involved in DT processes (Benedek and Fink 2019; Silvia 2015). In this context, working memory capacity and fluid intelligence are seen as necessary processes when working on DT tasks due to the ability to keep representations active and protect oneself from being distracted (e.g., Beaty et al. 2014; Engle et al. 1999). Fluid intelligence and executive functions are interrelated but not identical. Friedman et al. (2006) found a correlation between executive function *updating* (the ability to add to and delete information from the working memory) and fluid intelligence. In contrast, there was no correlation between fluid intelligence and executive functions *inhibition* (the ability to control mental operations) or *shifting* (the ability to switch between tasks), respectively. Diamond (2013) subsumed cognitive flexibility as part of the family of executive functions, which describe the ability to change perspectives and the ability to “think outside the box”. Flexibility, as assessed by DT tasks (Ionescu 2012) and switching in verbal fluency tasks (Nusbaum and Silvia 2011), can also be considered to reflect cognitive flexibility. Hence, it overlaps with creative thinking, task switching, and set-shifting (Diamond 2013). Diamond (2013) concluded that working memory, inhibitory control, and cognitive flexibility contribute to higher-order executive functions, namely *reasoning*, *problem-solving*, and *planning*, and that *reasoning*, as well as *problem-solving*, are identical with fluid intelligence. Taken together, DT might overlap with both executive functions and fluid intelligence. Benedek et al. (2012) specified that only certain types of intelligence are linked to certain aspects of creative thinking, and provided more differentiated insights into the relationship between these constructs. They found that *inhibition*, the ability to suppress irrelevant stimuli, was positively related to *fluency* (number of given responses) and *flexibility* (number of categories) of idea generation, whereas *originality*, reflecting the quality of ideas, was predicted by intelligence. Furthermore, Benedek et al. (2014b) presented evidence that while fluid intelligence and DT originality correlated moderately (*r* = .34), updating predicted fluid intelligence and to a lesser extent DT originality, whereas inhibition predicted only DT originality.

The focus on executive functions has recently increased due to research with neuroimaging methods (Silvia 2015). There is strong support for a top-down controlled view of cognitive processes in DT tasks. Idea generation appears to be a result of focused internal attention combined with controlled semantic retrieval (Benedek et al. 2014a). In addition, Frith et al. (2020) found that general intelligence and creative thinking overlap not only behaviorally (*r* = .63; latent variable correlation) but also in terms of functional connectivity patterns at the level of brain networks (i.e., 46% of connections were shared by networks that predicted either general intelligence or creative thinking). Importantly, this overlap of brain networks involved brain regions associated with cognitive control. It is expected that neuroscience research will pursue related lines of research to further unravel the neural basis of DT. In this vein, it seems that the interest of researchers regarding the relationship between intelligence and DT has expanded into different directions, including a more detailed view on intelligence facets and methodological considerations such as scoring methods or explicitness of instructions (Plucker and Esping 2015; Silvia 2015). We argue that a meta-analytical investigation of these potential moderators of the intelligence–DT correlation will help to clarify issues in the ongoing debate.

### 2.1. Moderators of the Relationship between Intelligence and DT

#### 2.1.1. Intelligence Facet

The CHC model (Carroll 1997; Horn and Cattell 1966; McGrew 2009) provides a useful framework to shed light on the link between DT and many cognitive abilities (Forthmann et al. 2019; Silvia 2015; Silvia et al. 2013). Based on the CHC model, intelligence can be distinguished between a higher-level general intelligence (g), a middle-level of broad cognitive abilities like fluid intelligence (g_f_), crystallized intelligence (g_c_), and a lower-level of narrow abilities. g_f_ reflects the ability to solve novel problems using controlled mental operations and includes inductive and deductive reasoning. In contrast, g_c_ reflects the declarative (knowing what) and procedural (knowing how) knowledge acquired in academic and general life experiences. Factor-analytic studies have shown that g_f_ and g_c_ load on the higher-level factor g (McGrew 2009). In this work, we explore the influence of intelligence facets on the intelligence–DT correlation.

#### 2.1.2. DT Instruction

Wallach and Kogan’s (1965) test battery includes an instruction to provide a playful environment with no time constraints to facilitate DT production. This *game-like* setting is recommended to reduce the impact of test anxiety or performance stress that could occur due to a test-like setting and could lead to overestimated correlations between intelligence and DT. However, a review of the studies applying this test revealed that many researchers ignore the *game-like* setting, probably for standardization and pragmatic considerations (e.g., regarding the amount of available testing time). Meanwhile, research has focused on the impact of clear and unambiguous instructions on the quality of DT production in a test-like setting (e.g., Forthmann et al. 2016; Nusbaum et al. 2014; for meta-analyses see Acar et al. 2020; Said-Metwaly et al. 2020). Even though many DT tests traditionally instruct participants to produce many ideas, researchers have begun to modify the instructions to be more specific about the test’s intention to work towards original ideas. For example, Forthmann et al. (2016) found a performance advantage resulting in a higher creative quality of ideational pools when participants were instructed to *be-creative*, compared to *be-fluent* instructions, which is in accordance with meta-analytical findings (Acar et al. 2020; Said-Metwaly et al. 2020). The meta-analysis of Kim (2005) did not differentiate between different settings (game-like vs. test-like), which is considered a limitation. To examine the impact on the relationship between intelligence and DT, in this meta-analysis, instructions were categorized into *be-fluent*, *be-original/be-creative*, *hybrid-fluent-flexible*, *hybrid-fluent-original*, *hybrid-flexible-original*, and *hybrid-fluent-flexible-original* (for the logic of hybrid instructions see (Reiter-Palmon et al. 2019)). In addition, *game-like* instructions vs. *test-like* settings were also coded, and it was expected that *game-like* instructions weaken the relationship between intelligence and DT.

Since there is no restriction in the *be-fluent* instruction, participants with a great amount of knowledge (g_c_) are deemed to benefit from the possibility to list any idea that comes to mind. In contrast, *be-creative* instruction requires the evaluation of whether upcoming ideas are original or not. In such conditions, participants who better control mental operations (g_f_) should improve their performance on DT tasks. Hence, the kind of instruction should interact with the intelligence facet and influence the relationship between intelligence and DT. Additionally, when receiving instructions that require participants to apply certain strategies to facilitate creative thinking (e.g., decomposition of objects in the Alternate Uses Task), DT performance is expected to correlate more strongly with intelligence as compared to instructions that do not imply such strategies (e.g., Nusbaum et al. 2014; Wilken et al. 2020).

#### 2.1.3. DT Scoring

It is crucial for scoring methods of DT tasks to consider at least the *originality* of responses to have a conceptual relation to the construct of creativity (e.g., Zeng et al. 2011), whereas, in the past, *fluency* (number of ideas) and *uniqueness* (frequency of occurrence in one sample) were common indicators for DT ability. It should be noted that *uniqueness* in some kind reflects the quality of the idea since unique ideas are at least not common ideas. However, ideas can be assessed as unique, even though they are not necessarily unusual, clever, original, or humorous (see overview in Silvia 2015). The confounding of *fluency* and *uniqueness* (more generated ideas increase the likelihood of unique ideas within the sample), the dependency of *uniqueness* on the sample size, and statistical aspects regarding the assessment of infrequency required adjustments (Silvia 2015). *Originality*, assessed by subjective ratings, provides a quality evaluation of the creative product but has its own weaknesses. *Originality* scorings have been critically discussed since researchers have applied varying scoring dimensions (i.e., novelty, unusualness, cleverness, overall creativity) and used different approaches (i.e., set ratings, top-scoring; for a review see Reiter-Palmon et al. 2019). However, research has provided mixed results regarding the relationship between fluency and subjective ratings of originality/creative quality (Forthmann et al. 2020b; Plucker et al. 2011; Silvia 2015).

DT outcomes can be distinguished into quantitative (*fluency*, *flexibility*, *elaboration*) and qualitative (*originality* or any other *creative quality*) measures, and the type of scoring affects the relationship between intelligence and DT. Batey and Furnham (2006) found a smaller correlation when DT was assessed by *originality* than *fluency* scoring methods. However, since many researchers have recommended explicit *be-creative* instruction (Chen et al. 2005; Nusbaum et al. 2014), the focus in this meta-analysis lies in the interactional effects of DT outcome, instruction, and intelligence facets.

Silvia (2015) postulated that the access, manipulation, combination, and transformation (g_f_) of the knowledge (g_c_) is the key to DT. Hence, the involvement of g_f_ is supposed to have a substantial impact on the relationship between intelligence and DT. However, Silvia and colleagues conceptualized DT tests with a *be-creative* instruction, used subjective scorings to evaluate the outcome, and recommended the correction for measurement errors (i.e., Nusbaum and Silvia 2011; Silvia 2015). Therefore, it is hypothesized that the combination of instruction (*be-creative*), DT outcome (*originality*), and correction for measurement error increases the involvement of g_f_ in DT and, hence, the relationship between intelligence and DT.

#### 2.1.4. Time on Task

Time on task was found by Preckel et al. (2011) to influence the relationship between intelligence and DT strongly. They found a stronger correlation between intelligence and DT when both were assessed under rather speeded conditions (i.e., around 2 min on task) compared to unspeeded conditions (i.e., around 8 min on task). Notably, the stronger relationship under speeded conditions was driven by shared variation of both measures with mental speed. Divergent thinking in Preckel et al. (2011) was assessed with be-fluent or be-fluent-be-flexible hybrid instructions and, hence, we expected a stronger correlation for these conditions when time-on-task is short (i.e., speeded conditions) for intelligence and DT (i.e., interaction of time-on-task for both measures). However, recent research by Forthmann et al. (2020a) suggests that such a result is not expected when DT is assessed with be-creative instructions and scored for creative quality. It is further noteworthy that timed testing implies a vital role for typing speed in DT assessment (e.g., Forthmann et al. 2017). For instance, participants may be able to think of more ideas than they can type when time is limited (e.g., single-finger typists) or because they type slowly, they have ideas that get blocked or are not recorded.

#### 2.1.5. Intelligence Level

One of the aims of Kim’s meta-analysis was to find evidence for the threshold hypothesis, which states that there is a positive relationship between intelligence and DT for people with an intelligence quotient (IQ) lower than a certain threshold and vanishes or becomes statistically non-significant once the IQ exceeds the threshold (most often an IQ of 120 is assumed as a threshold; e.g., Jauk et al. 2013; Karwowski et al. 2016a). Previous tests of the threshold hypothesis provided mixed results, yet several studies were plagued with inconsistent decisions regarding the criteria for support or rejection of the threshold hypothesis and concrete analytical decisions on how to test it (e.g., see Karwowski and Gralewski 2013, for a discussion). In Kim’s meta-analysis correlations of *r* = .235 and *r* = .201 for IQ below and above 120, respectively, did not differ statistically. Further analyses with four IQ levels (i.e., IQ < 100, IQ ranging from 100 to 120, IQ ranging from 120 to 135, and IQ > 135) produced mixed results. Thus, the threshold hypothesis was not confirmed. It would be possible to revisit this hypothesis but treat intelligence as a continuous moderator variable to avoid choosing a certain threshold a priori (see for a discussion Karwowski and Gralewski 2013; Weiss et al. 2020b). However, based on average IQ in different samples, it cannot be ruled out that parts of the IQ distributions overlap, which highlights the importance to take the different level of analysis as compared to primary studies into account. That is, splitting the sample according to a predefined threshold yields groups of participants with disjunct ranges of measured IQs. However, meta-analysis operates at the level of effect sizes, and differences in sample means of IQ do not imply that participants from such studies have non-overlapping IQ ranges (i.e., average IQ is only a rough proxy). Consequently, we believe that the methodology of meta-analysis is not well suited to examine the threshold hypothesis, but such investigations require focused analytical approaches (see Jauk et al. 2013; Karwowski et al. 2016a; Weiss et al. 2020b), and therefore do not reinvestigate threshold hypothesis in this meta-analysis.

#### 2.1.6. Modality of Tasks

DT and intelligence tasks differ in terms of the modality of the item content. DT tasks are most often studied in the verbal domain, at least when older children, adolescents, and adults participate. However, figural and numerical DT tasks exist as well (e.g., Preckel et al. 2011). Sometimes a composite score for DT based on tasks from several modalities is derived and used. The same variety of modalities exist for intelligence measures. We explored task modality’s influence on the intelligence–DT correlation and assumed that effect sizes should be largest when DT and intelligence modality are congruent.

### 2.2. Aim of the Current Work

The aim of the current work is to update Kim’s (2005) meta-analysis by including recent work in this field and to consider additional moderators based on recent theorizing that were not taken into account in her work, such as intelligence facet, DT instruction, and time on task (all in relation to DT scoring). In addition, we aimed at correcting for attenuation (i.e., measurement error), modeling the clustering of the effect sizes (i.e., correlations are nested in articles), and examining publication bias. In relation to this, it should be noted that correcting for attenuation and correcting for measurement error within latent variable frameworks are not the same (e.g., Borsboom and Mellenbergh 2002). Given that latent variable modeling approaches were expected to be used far less often, we chose correction of attenuation to take measurement error into account (of course, when primary data allow for latent variable modeling, it is preferred over correction for attenuation; see Borsboom and Mellenbergh 2002). Finally, our analysis strategy accounted for the confound of DT scores by response fluency (e.g., Forthmann et al. 2020b).

## 3. Materials and Methods

### 3.1. Eligibility Criteria

The identification of potential studies to be included in the meta-analysis was based on a literature search in relevant databases. Moreover, different criteria had to be fulfilled by these studies. Only empirical studies in English or German language from journal articles, PhD theses, books, or DT test manuals were considered. Studies needed to provide correlation coefficients (or other measurements from which correlation coefficients could be computed) of intelligence–DT measures, detailed information on DT testing procedures and DT scoring, and at least the information on the applied intelligence test. We only considered effect sizes from studies that employed established DT tests (i.e., not verbal fluency) and established intelligence tests (i.e., not proxy measures such as school achievement). Moreover, creative production tasks that typically ask for single products per task such as metaphor and humor production tasks, for example, were also not eligible. We further included only works in which intelligence measurement was based on either full test batteries, including many different task types, or reasoning tasks, or vocabulary and knowledge tasks to have clear measures of g, g_f_, and g_c_, respectively.

Finally, all data were derived from healthy participants. No general restrictions were made regarding the publication date of the study or the participants. Sources were retrieved until June 2019.

### 3.2. Literature Search

**Figure 1:**
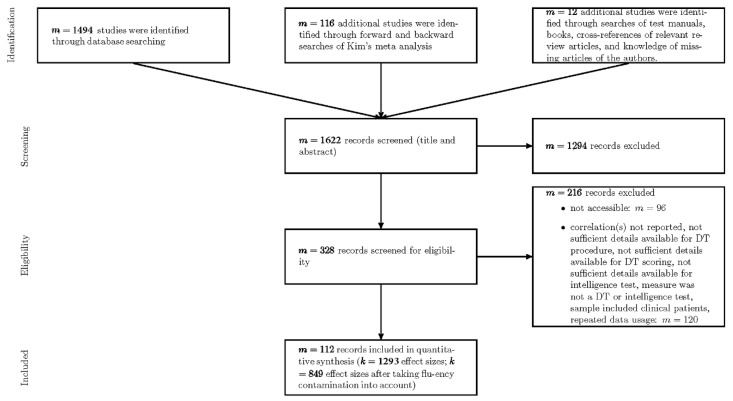
Illustration of the applied search and selection process.

Computer search was conducted in the databases Academic Search Premier, PsychARTICLES, Psych Critiques, PsychINFO, and PSYNDEX with five search terms. The search terms were divided into two parts. The first part included search terms for DT; the second part included search terms for intelligence. Both parts were linked by an AND connection. DT search terms were either general (divergent thinking) or represented specific DT test names (Alternate Uses Test, TTCT) and names of prominent DT researchers (Guilford, Wallach and Kogan, Torrance). The intelligence search term included IQ, intelligence, cognitive and mental abilities. The applied search terms can be found in the Open Science Framework (OSF; https://osf.io/s4hx5). The search resulted in 1494 studies, covering the years from 1962 to 2019. Forward and backward searches of the meta-analysis of Kim (2005) yielded 116 additional results. Five test manuals with validation data, one book, and one article were included after further search. Six studies were identified by cross-references of review papers on the topic or based on knowledge of missing works of one of the authors of this work. By screening titles and abstracts, 328 potentially relevant studies were identified. Out of these, 232 studies were accessible for more extensive review. This review yielded 112 records that met all eligibility criteria. From the *k* = 1293 obtained coefficients, *k* = 849 (65.66%) were retained for analysis after excluding coefficients affected by fluency contamination. Importantly, a substantial amount of 67 studies were published after the release of Kim´s meta-analysis in 2005, emphasizing the relevance of a meta-analytic update. The applied search procedure is illustrated in the flow-chart in Figure 1 following PRISMA guidelines (Moher et al. 2009). It should be noted that works falling within the publication years from 1962 to 2016 were identified, screened, and checked for eligibility by the first author. For this period, we did not specifically document the frequencies of each different exclusion reason. In the course of updating the database, all articles in the publication years from 2017 to 2019 were screened (for more details see Section 3.3). Then, the second author checked all accessible works for eligibility. For this eligibility check, specific exclusion reasons are available and we uploaded the eligibility check file to the OSF page of this project (https://osf.io/s4hx5). We also provide a list with all included studies at the OSF repository.

### 3.3. Coding Procedure

Study information (authors, title, year of publishing, country, and publication type) and sample information (sample size, mean age, *SD* of age, gender (i.e., we coded the number of female participants to finally calculate a gender ratio score that reflects the relative frequency of females in a sample), school grade, sample type, mean IQ, *SD* of IQ) was initially coded. A total of 1186 studies (publication year of these studies ranged from 1962 to 2016) was screened and coded by the first author. Disputable cases were resolved in a discussion by the first and third authors. Another 436 studies (publication year of these studies ranged from 2016 to 2019) were screened by three student assistants and coded by the second author. Finally, the second author screened all coded studies for inconsistencies which were resolved based on discussions by the second and third authors. Reliabilities of DT and intelligence tests were coded to provide the opportunity to adjust correlation coefficients for more reliable results (Schmidt and Hunter 1996). Since reliabilities were not reported in all studies, missing reliability estimates for DT and intelligence were imputed by mean reliability (Schmidt and Hunter 2015). While this is a pragmatic approach, we argue that it is still more realistic than assuming perfect reliability of the measures. The imputed values for reliability were .78 and .79 for DT and intelligence, respectively. The coding scheme is openly available in the OSF repository: https://osf.io/s4hx5. The coding scheme is accompanied by a table that includes a list of all coded DT and intelligence measures.

#### DT Coding

We coded the name of the DT test (e.g., Torrance Test of Creative Thinking, Wallach-and-Kogan Tests) and the name of the task type (e.g., Alternate Uses, Line Meanings, Consequences). In addition, we coded the modality of the DT task (verbal, figural, or several), instruction of the DT task (i.e., be-fluent, be-original/creative, hybrid-fluent-flexible, hybrid-fluent-original, hybrid-flexible-original, or hybrid-fluent-flexible-original), other aspects of instructions (e.g., game-like, test-like, or specific strategy instructions), time condition (i.e., untimed, timed at the item-level, or timed at the test level), and time on task in minutes when the information at the item-level was available.

In addition, DT scoring information was categorized into six different categories: *fluency*, *flexibility*, *elaboration* (e.g., number of used words to describe the idea), *originality/creative quality* (creative quality can be understood as any scoring that has a conceptual relation to a definition of creativity; see (Forthmann et al. 2017)), *the composite score* (sometimes referred to as the creativity index), and *other* scorings. In case different DT outcomes were combined, be it through averaging or summing up scores, they were categorized as a *composite score*. DT outcomes that did not fit into any of the main categories (e.g., abstractness of titles) were classified as *other*. Effect sizes for the other category were omitted due to the presence of only one study and *k* = 18 effect sizes.

We addressed the confounding effect of fluency by employing the following strategy: (a) We coded if a correlation was based on a confounded score (i.e., summative aggregation; see (Forthmann et al. 2020b)); (b) when fluency was scored, all other summative scores of the same study were excluded from analysis; (c) if fluency was not available, a flexibility score was chosen first to reflect a quantitative measure; and (d) if fluency and flexibility were not available, a summative originality score was chosen first to reflect a quantitative measure. As a consequence of this procedure, 444 correlation coefficients (34.34%) were excluded from analyses to prevent fluency contamination of the results.

### 3.4. Statistical Procedure

The meta-analysis was conducted using the metafor package for R (R Core Team 2016; Viechtbauer 2010). To compare data, correlation coefficients were transformed to Fisher *z* metric using the formula:

(1) $$z=\frac{1}{2}\ln\left(\frac{1+r}{1-r\;}\right).$$

Sample variance was estimated by weighting the sample size with

(2) $$v=\frac{1}{\left(n-3\right)}.$$

Most coefficients were clustered within studies since many studies reported several correlations from the same sample. To control for dependent data, a three-level meta-analysis was computed (Konstantopoulos 2011). This statistical method splits the variance into sampling variance and two sources of true variance: the between-study variance, accounting for the variability between the studies (i.e., [average] effect sizes between studies vary and are assumed to have a distribution of true effect sizes), and the within-study variance, accounting for the variability within the different studies (i.e., effect sizes within studies vary across measures; e.g., intelligence facets). As a result, estimates of standard errors are unbiased and more robust. True variance was estimated using the restricted maximum likelihood estimator (REML) to avoid bias or underestimation of the variance due to less reliable estimators (Viechtbauer 2005). Meta-regressions based on categorical moderators were computed with cell mean coding to facilitate interpretations of the results. Subsequently, a series of theoretically guided linear hypothesis tests were applied to test the differences of the estimated effects for the various combinations of moderator categories. For each of the tested set of contrasts we applied a Bonferroni–Holm correction to the respective *p* values. We tested these moderators in separate analyses. This approach further requires checking of possible confounding of moderators which may lead to wrong conclusions (Viechtbauer 2007). While separate analyses retain most of the available effect sizes, building meta-regression models that include all moderators at the same time reduces the number of included effect sizes heavily when not all moderators can be coded for all effect sizes. We addressed this issue by a robustness check of our moderator findings (only when a moderator test was significant) in Section 4.3.12. Please note that we report only moderator effects that were found to be robust across these checks when reporting results for separate analyses. Detailed patterns of which effects were found only for specific analytical setups are reported in Section 4.3.12. The effect sizes were adjusted applying the attenuation formula (Schmidt and Hunter 2015),

(3) $$r_{\left(adj\right)}=\frac{r}{\left(\sqrt{Reliability_{DT}}\;*\;\sqrt{Reliability_{Intelligence}}\right)}$$

and then transformed into Fisher *z* metric. All results described in the next section are reported in retransformed Pearson´s correlation

(4) $$r=\frac{\exp\left(2z\right)-1}{\exp\left(2z\right)+1}.$$

## 4. Results

Data involved 849 coefficients from 112 studies (*N* = 34,610). The age of the participants across samples ranged from 4.00 to 72.93 years (*M* = 20.40, *SD* = 12.20). Most of the samples comprised of school students (40.50%), followed by university students (34.71%), and adults (15.70%). Other sample types were preschoolers (4.13%) or mixed samples of any of the previously mentioned sample types. The average gender ratio across samples was 0.55 (*SD* = 0.26) with a range from 0 (only male participants) to 1 (only female participants). The average sample size was 274.68 (*SD* = 615.28) with sample sizes ranging from *N* = 10 to *N* = 5337 participants. Most studies were from the USA (*m* = 51; UK: *m* = 12; Germany: *m* = 9; Austria: *m* = 8; other countries contributed up to 3 studies: Australia, Canada, Chile, China, France, Hungary, India, Israel, Italy, Korea, Lebanon, Netherlands, New Zealand, Norway, Philippines, Poland, Romania, Russia, Singapore, Spain, Taiwan, and United Arab Emirates).

The most frequently used DT task was the Alternate Uses Task (33.80% of effect sizes). Next, 30.62% of the effect sizes were based on a DT test battery (e.g., TTCT, ATTA, Wallach-and-Kogan, EPoC, BIS, VKT, and so forth). Moreover, 13.66% effect sizes were based on a composite of several DT tasks (i.e., a test battery created in an ad hoc fashion for research purposes). Other DT tasks that were used for 2% to 5% of the effect sizes were Pattern Meanings, Line Meanings, Instances, Similarities, and the Consequences task. The most frequent task modality for DT was verbal (70.20% of effect sizes), 19.55% of the effect sizes were based on figural DT tasks, and only 10.25% of the effect sizes relied on several task modalities. The most frequent intelligence test was one of the variants of the Wechsler test (17.83% of the effect sizes), followed by one of the variants of the IST (9.60%; German: *Intelligenzstrukturtest*; translates as Intelligence-Structure-Test) and a variant of the Raven’s (7.13%). Many other tests were used to measure intelligence and the interested reader has access to the fully coded data at the OSF repository (https://osf.io/s4hx5). Clearly, the used intelligence measures were far more heterogeneous as compared to the used DT measures. Moreover, task modality was more evenly distributed for intelligence measures with 36.98% of the effect sizes based on verbal, 28.86% based on figural, 27.33% based on several modalities, and 6.83% based on numerical intelligence measures.

### 4.1. Overall Effect

The estimated overall correlation between DT and intelligence was *r* = .25, 95%-CI [.21, .30] (*m* = 112, *k* = 849). It was further revealed that both the variance components for between-study variation (χ^2^(1) = 161.00, *p* < .001) and within-study variation (χ^2^(1) = 2758.15, *p* < .001) of effect sizes were needed in the multi-level model. A great amount of heterogeneity (*I*^2^ = 92.07%) could be accounted for by true effect size variance, with slightly more variance within ($I_{w}^{2}$ = 46.58%) than between studies ($I_{b}^{2}$ = 45.49%). That is, variation of effect sizes quantifying the correlation between DT and intelligence within the studies (e.g., when correlations are based on different measures of intelligence or DT) and between-study variation (i.e., when effect sizes are aggregated at the study level) were highly comparable. As expected, obtained effects were heterogeneous, *Q_E_*(848) = 9206.17, *p* < .001.

### 4.2. Publication Bias

**Figure 2:**
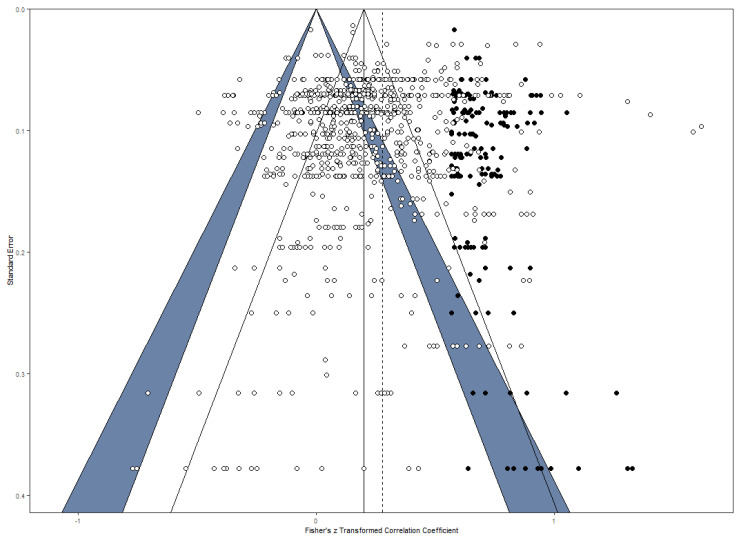
Funnel plot to test for publication bias. Effect sizes are depicted as Fisher-*z*-transformed correlations. Light dots represent empirical data, dark dots represent imputation data according to the trim and fill method. Shaded regions refer to significance contours (Peters et al. 2008).

A publication bias occurs when studies reporting significant or desired results are preferred for publication, while studies reporting insignificant results are more likely to be rejected. As a result, the accessible data does not represent all results of scientific studies and might over- or underestimate certain effects (Sterling et al. 1995). To control for possible publication bias, as a first step, a funnel plot (see Figure 2) was used as a visual method to examine the distribution of the coefficients (Duval and Tweedie 2000). The distribution for all effect sizes appeared to be asymmetric. In the next step, the number of missing coefficients was estimated by the trim and fill method (Duval and Tweedie 2000) and imputed 169 data points on the right side of the funnel plot (see Figure 2). All of these imputed values were found in the region of significant or highly significant effect sizes which implies that the mechanism underlying the asymmetry was not a classical publication bias based on the publication of only significant findings (Peters et al. 2008). Next, an Egger-type test was computed (i.e., the standard errors of the correlation coefficients were used as a moderator in the three-level model) and confirmed the publication bias (*z* = −2.37, *p* = .018). This publication bias suggests that the effect is underestimated. When taking the 169 imputed effect sizes into account, a significant correlation of *r* = .27, 95% CI [.25, .29], *p* < .001, appears, indicating that the true correlation was only slightly underestimated because of publication bias (recall that the overall estimate reported above was found to be *r* = .25). However, it should be mentioned that the analysis with imputed data points did not take into account the within-study variance and was hence conducted by using a random effects model providing two sources of variance: sampling variance and true parameter variance. We repeated the publication bias analysis when aggregating effect sizes at the level of independent samples as a check of robustness. For these aggregated data, the same pattern of asymmetry was found (see OSF repository for detailed findings).

### 4.3. Moderator Analysis

#### 4.3.1. Bivariate Relationships of Moderators

Prior to moderator analysis, we investigated all bivariate relationships between moderators to check for potential confounding effects between moderator variables (Viechtbauer 2007). We calculated Pearson correlations for pairs of continuous moderators, the η coefficient for pairs of continuous and categorical moderators, and Cramer’s *V* for categorical moderators. All correlations are reported in Table 1.

As a first observation, the publication year correlated with several other moderators such as time-on-task for intelligence measures, DT instruction, DT scoring, DT time condition, and intelligence time condition. In particular, the correlations with aspects of how DT and intelligence are measured reflect changes of methodological approaches over time. Applications of be-creative instructions (vs. be-fluent instructions), originality/creative quality scoring (vs. fluency), and timed DT testing conditions (vs. untimed conditions) have increased over the years. The pattern for timed DT testing conditions was found to be reversed for intelligence testing conditions (untimed testing of intelligence increased as a function of publication year). Several other bivariate correlations were rather strong. For example, DT time-on-task correlated strongly with DT modality because of the common practice to use longer testing times when drawing is required for figural DT task as compared to verbal DT tasks. Similarly, intelligence facet and modality were strongly correlated because g_f_ is hard to measure in the verbal domain and relies most often on figural item content. All these observations need to be taken into account when interpreting the reported moderator analyses below. Please note further that we take confounded moderators into account in a robustness check (see Section 4.3.12). The found bivariate associations further highlight the complexity when examining effect sizes from primary studies as the unit of measurement.

#### 4.3.2. Intelligence Facet

The estimated correlations between DT and intelligence facets were found to be small to moderate and in the range from .23 to .28 across g, g_c_, and g_f_ (see Table 2). The moderator test was non-significant (*Q_M_*(2) = 5.77, *p* = .056). Given the borderline significant moderator test, it was considered worthwhile to examine specific contrasts here. There was no difference between any two of the intelligence facets (all *p*s > .161).

#### 4.3.3. DT Instruction

Table 3 displays the correlation estimates between DT and intelligence as a function of the used DT instruction. The moderator test was non-significant, *Q_M_*(6) = 2.63, *p* = .854. Importantly, most studies relied on be-fluent instructions (i.e., 59 studies), and only fourteen studies used be-original/creative instructions.

We further analyzed other aspects of DT instructions, such as the classical comparison between “game-like” vs. “test-like” conditions, and added concrete strategy instructions as another aspect of DT instructions. The results for this moderator analysis are displayed in Table 4. Game-like DT instructions yielded a non-significant correlation between DT and intelligence, whereas effect sizes were small to moderate for test-like or strategy DT instructions. The moderator test was significant, *Q_M_*(2) = 25.94, *p* < .001. Specific contrasts revealed that the correlation was significantly higher for test-like DT instructions (*z* = −4.94, *p* < .001) and strategy DT instructions (*z* = −3.96, *p* < .001) as compared to game-like DT instructions, respectively, whereas test-like and strategy DT instructions did not differ (*z* = 1.21, *p* = .226). These specific contrasts were mostly robust across both checks (see Section 4.3.12). However, the contrast between strategy instructions and game-like instructions was only significant by trend (*p* = .099) when including additional moderators in the regression model (see Section 4.3.12).

#### 4.3.4. DT Scoring

DT scoring was found to be a significant moderator of the relationship between DT and intelligence, *Q_M_*(2) = 22.47, *p* < .001, with correlations ranging between *r* = .20 (fluency) to *r* = .37 (composite score; see Table 5). Specific contrasts further revealed that DT fluency scores correlated significantly lower with intelligence as compared to originality/creative quality (*z* = −3.75, *p* < .001). This latter finding was robust across both checks, but the contrast found between DT fluency and composite scores was not robust across the checks (see Section 4.3.12). Hence, the size of the correlation for DT composite scores can be partially explained by confounding with other moderators.

#### 4.3.5. DT Instruction-Scoring-Fit

To assess instruction-scoring fit, the dataset was restricted to be-fluent and be-original/creative instructions and fluency and originality/creative quality scorings for DT. As expected, a model including the interaction between instruction and scoring showed a significantly better fit to the data, χ^2^(1) = 6.70, *p* = .010 (vs. a model including only main effects). As expected, the highest correlation was found for DT be-original/creative instructions when DT was scored for originality/creative quality (see Table 6). This correlation was significantly higher as compared to the combination of be-fluent instructions and fluency scoring (*z* = −3.16, *p* = .008), and the combination of be-original/creative instructions and fluency scoring (*z* = −4.88, *p* < .001). Interestingly, under be-fluent instructions, the correlations of intelligence with DT fluency and DT originality did not differ (*z* = −0.94, *p* = .697). All other contrasts were non-significant (all *p*s > .052). These findings were robust across the checks (see Section 4.3.12). It should further be noted that the contrast between be-fluent-originality vs. be-creative-originality was at least significant by trend across robustness checks.

#### 4.3.6. Interaction of DT Instruction-Scoring-Fit and Intelligence Facet

Next, intelligence facet was added to the examination of instruction-scoring fit. A model including the three-way interaction between instruction, scoring, and intelligence facet did not improve model fit beyond a model, including the *Instruction × Scoring* interaction and the main effect of intelligence facet, χ^2^(6) = 2.78, *p* = .836.

#### 4.3.7. Time on Task

As an initial step, we compared if testing was timed or not. We proposed an interaction effect between the timing of DT and timing of intelligence when a be-fluent instruction and scoring for fluency was used for DT measures. This interaction was not straightforwardly testable because we did not find any studies in the literature in which both DT and intelligence were tested in untimed conditions. Hence, we aimed at examining all correlation coefficients available when crossing the moderators: instruction (restricted to be-fluent and be-original/creative instructions) and DT scoring (restricted to fluency and originality/creative quality). However, there were not enough effect sizes available for the cells of the targeted interaction effect involving untimed tasks and, hence, the proposed interaction could not be tested in a reasonable way.

Next, we focused on time-on-task in minutes (i.e., effect sizes based on untimed assessment conditions were excluded) and tested the interaction between time-on-task for DT and intelligence (be-fluent and fluency: *k* = 45, *m* = 15; be-creative and originality/creative quality: *k* = 109, *m* = 8) and found it to be non-significant (both *p*s > .674). Moreover, for both combinations of instruction and scoring none of the main effects of time-on-task was significant (all *p*s > .405).

#### 4.3.8. Task Modality

Task modality varied at the level of DT tasks and intelligence tasks. First, we checked if an interaction between DT modality and intelligence modality improved model fit beyond a simple main effect model, which was not the case (χ^2^(6) = 1.68, *p* =.947). However, it was found that figural DT measures correlated significantly less strong with intelligence measures as compared to verbal DT measures (β = −0.10, *z* = −4.20, *p* < .001). However, this observation was not robust across all checks (see Section 4.3.12) and should be interpreted with caution. Given the theoretical and empirical relationship between intelligence task-modality and intelligence facet (i.e., g_c_ will most likely be measured by verbal tasks, whereas g_f_ will most likely be measured by figural tasks), the same models were tested when substituting intelligence task-modality by intelligence facet (e.g., g_c_ measures should correlate strongest with verbal DT). However, also for this slightly different combination of variables no interaction was found (χ^2^(4) = 2.02, *p* =.732). To further test the proposed modality-congruency effect, a variable was constructed to contrast correlations based on non-congruent modalities (*k* = 507, *m* = 83, *N* = 25,131) with correlations based on congruent modalities (*k* = 342, *m* = 67, *N* = 17,610). The intelligence–DT correlation was not found to be stronger for congruent modalities of measures (β = 0.03, *z* = 1.58, *p* = .115). For completeness, we report all estimated correlation coefficients for all combinations of DT modality and intelligence modality in Table 7.

#### 4.3.9. DT Task-Type

DT task-type was found to be a significant moderator of the intelligence–DT correlation, *Q_M_*(8) = 23.96, *p* < .001 (see Table 8). However, none of the specific contrasts reached statistical significance (all *p*s > .052). Additionally, robustness checks did not suggest any differences between DT task-types.

#### 4.3.10. Comparison of Pre-Kim and Post-Kim Effect Sizes

Publication time was taken into account by coding if studies were published in 2004 or earlier (i.e., the search scope of Kim’s meta-analysis) vs. published later than 2004. Given that Kim did not correct for attenuation of correlations, we first compared pre-Kim and post-Kim effect sizes at an uncorrected level. This contrast was non-significant (*β* = 0.00, *z* = 0.10, *p* = .921). The uncorrected correlation for the time period of Kim’s meta-analysis was *r* = .19, 95%-CI: [.14, .23], which nicely covers Kim’s estimate of *r* = .17. The sample size for coefficients included from 2004 or earlier was *N* = 9581 (*k* = 483, *m* = 45) and coefficients taken from research published later than 2004 were based on *N* = 25,029 (*k* = 366, *m* = 67). The same contrast for the analysis corrected for unreliability was also non-significant (*β* = −0.01, *z* = −0.30, *p* = .766). The correlation for Kim’s time period corrected for attenuation was *r* = .26, 95%-CI: [.20, .32].

#### 4.3.11. Sample Characteristics

**Figure 3:**
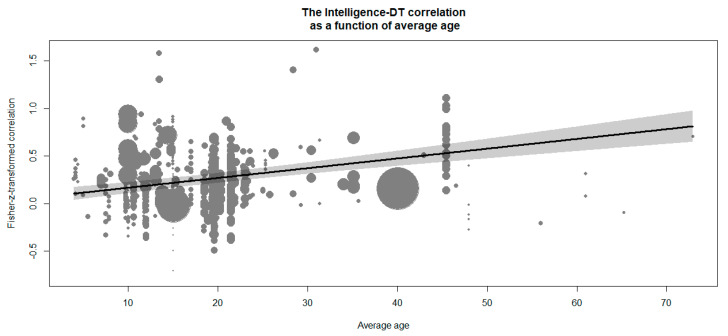
Relationship between Fisher-z-transformed intelligence–DT correlations (*y* axis) and average sample age (*x* axis). The size of the dots reflects the weight received in the meta-regression model. The linear regression slope is depicted in black along with a 95% confidence band (gray area around the regression slope).

Finally, we tested the mean age and gender ratio as the most common sample characteristics. The average sample age had a positive relationship with the intelligence–DT correlation, *β* = 0.01, *z* = 6.93, *p* < .001. This effect implies that a 10-year difference in average sample age yields a difference of .10 in the Fisher-z-transformed intelligence–DT correlation. This effect is visualized in Figure 3. Importantly, a quadratic relationship of average age and transformed intelligence–DT correlations did not improve model fit, Δχ^2^(1) = 0.09, *p* = .762, and the linear effect was robust across both checks (see Section 4.3.12).

**Figure 4:**
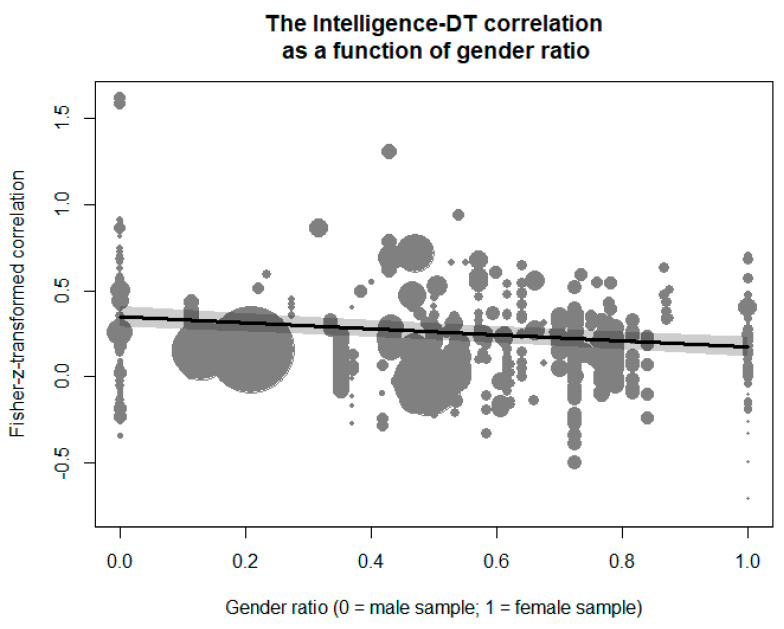
Relationship between Fisher-z-transformed intelligence–DT correlations (*y* axis) and gender ratio (*x* axis). A gender ratio of 0 implies no females in the sample, whereas a value of 1 implies no males in the sample. The size of the dots reflects the weight received in the meta-regression model. The linear regression slope is depicted in black along with a 95% confidence band (gray area around the regression slope).

In addition, gender ratio had a negative effect on the transformed intelligence–DT correlation (see Figure 4), *β* = −0.17, *z* = −5.09, *p* < .001. The meta-regression implied that the correlation for samples comprising completely of males was *r* = .34, 95%-CI: [.28, .39], whereas a sample comprising completely of females would yield a correlation of *r* = .17, 95%-CI: [.12, .23]. A sample with a uniform distribution of gender would further imply a correlation of *r* = .26, 95%-CI: [.21, .30]. Notably, gender ratio was not found to be strongly confounded by any of the other moderators (i.e., none of the bivariate correlations with gender ratio was >.40; see Table 1), and the robustness check in which all other moderators correlating >.20 with gender ratio were added to the meta-regression revealed a highly comparable pattern.

#### 4.3.12. Robustness Check

We applied robustness checks to further test the dependability of the above reported moderation results. In a first step, we re-examined each moderator effect that reached significance in a meta-regression model that additionally included all other moderators that correlated > .40 with the moderators under consideration (see Table 1). In a second step, we reran the analysis now including all moderators that correlated > .20 in the confounding check. Results of the robustness checks are presented in Table 9. It shows that most but not all moderation effects provided to be at least fairly robust. For better readability of the results section, we refer above only to those effects that passed the robustness checks and also explain non-robust findings along with the findings from separate moderator analyses. Complete results for all checks can be found in the OSF repository (https://osf.io/s4hx5).

## 5. Discussion

The aim of this meta-analysis was to provide an update on the relationship between intelligence and DT by investigating the role of different DT scoring methods, task instruction, and a more specific view on intelligence facets beyond other previously established moderators. The overall effect indicates that the relationship is small to moderate (*r* = .25, 95%-CI: .21, .30) and, hence, slightly (but significantly) larger as compared to prior findings of Kim´s meta-analysis (2005; *r* = .17, 95%-CI: .17, .18). This difference for the overall correlation can be fully attributed to corrections for attenuation because a difference between pre-Kim and post-Kim effect sizes was not found regardless of whether correction for attenuation was applied or not. Nevertheless, it can be observed that studies published after 2004 report reliability measures more frequently (and corrected for measurement error more often). Hence, the studies’ methodological quality might have increased in recent years, but not necessarily the overall relationship between various measures of intelligence and DT. Moreover, moderation analyses showed that the intelligence–DT relationship is, however, somewhat higher (up to *r* = .31–.37) for specific test conditions such as when employing test-like, timed assessments, using be-creative instructions, and considering DT originality scores.

Following recent propositions and empirical findings (Silvia 2015), we expected that g_f_ correlates stronger with DT as compared to the correlation between g_c_ and DT. This contrast was particularly expected for be-creative instructions and originality/creative quality scorings. However, when participants are instructed to *be-creative*, the correlation between DT and g_c_ and DT and g_f_ was quite similar. Notably, the usually high correlation between g_f_ and g_c_ (*r* ≈ .60–.70; e.g., Bryan and Mayer 2020) could preclude finding a differential pattern for these intelligence facets. Hence, it seems that g_c_, namely knowledge, is required as a basic prerequisite and that g_f_ is required to form and evaluate ideas when participants are explicitly instructed to be creative. In addition, the fact that g_c_ and DT are correlated indicates that knowledge is required for DT, which supports the historical view of Mednick (1962). This knowledge could be used to simply recall relevant solutions (e.g., Miroshnik and Shcherbakova 2019), or provide the conceptual elements to be combined to original ideas. It must not be overlooked that by asking participants to produce many ideas, a DT test resembles verbal fluency tasks (Nusbaum et al. 2014). Beauducel and Kersting’s (2002) view that verbal fluency tasks are markers for g_c_ would have suggested that DT and g_c_ correlate more strongly (as compared to the DT–g or DT–g_f_ correlations) when be-fluent instruction and fluency scoring are used to assess DT. However, this specific proposition was not supported in this study.

The correlations did not generally differ whether a *be-fluent* or a *be-creative* instruction (or any other variant of a hybrid instruction) is given (i.e., we did not find a main effect of instruction). However, correlations increased for the *be-creative* instruction when DT responses were scored for *originality* (i.e., in case of instruction-scoring fit) compared to *fluency* scorings when be-fluent instructions were used. This pattern has been expected by Silvia (2015). Be creative instructions are thought to induce more cognitively demanding strategies and thus increase the relevance of intelligence and executive control. In addition, the intelligence–DT correlation was also found to be stronger for the combination of be-creative instructions and originality scoring as compared to be-creative instructions and fluency scoring. Clearly, the interaction of instruction and DT outcome has an impact on the relationship between intelligence and DT. Instructing participants to *be-creative* leads to more sophisticated responses, whereas instructing participants to *be-fluent* influences the number of responses. Hence, researchers get what they ask for (Acar et al. 2020). Since DT is considered to be a marker of creative potential (Runco and Acar 2012), the assessment of the *originality* of the responses seems required (e.g., Zeng et al. 2011) and, as a matter of fact, is provided in most of the recent studies. All scoring methods have their weaknesses, and the development of new methods (e.g., based on corpus semantics; Beaty and Johnson 2020; Dumas et al. 2020) continues, and we can be curious about how the weaknesses of the current methods will be mitigated.

Correlations in *game-like* instructions dropped to a small effect size that was non-significant. Compared to the test-like settings (or strategy instructions), it seems that under *game-like* instructions, the relationship between intelligence and DT diminishes. However, it remains unclear to what extent the testing situation influences the relationship between intelligence and DT. Affective/conative factors like current motivation, time pressure, or test anxiety may play a major role when assessing DT. For example, Byron et al. (2010) ran a meta-analysis on the relationship between stressors and creativity and found that when participants expect an evaluation, their creative performance is influenced in an inverted U-shaped manner. Strong evaluative stress or the absence of an evaluation of the performance hinders creative performance, whereas minor levels of evaluative stress support creative performance. Test situations imply the evaluation of the outcome and might be responsible for better performance. Since a considerable part of the participants are students, it can be assumed that they are familiar with test settings and that the expected evaluation of their results may have motivated them to give their best. On the other hand, the evaluative component is absent in a *game-like* setting and may hinder creative performance. In fact, playing a game does not necessarily stimulate the motivation to perform well. Furthermore, there were no time-constraints for the tasks in the *game-like* setting. What is more, game-like conditions can also drastically vary from study to study. Thus, not all studies with game-like conditions rely on the same approach (see Said-Metwaly et al. 2017). In a study with adolescents, Preckel et al. (2011) found stronger correlations between reasoning tasks and DT, when DT was assessed with time constraints compared to the test without time limits. Further, Beaty and Silvia (2012) found that more ideas are generated at the beginning of a DT task, whereas originality increased with time. Hence, the allowed time to work on a task might play a crucial role in creative performance. However, whether the game-like setting or accompanying aspects can solely account for the missing relationship between intelligence and DT remains an open issue. Future research projects should consider that the relationship between intelligence and DT may be biased due to the environmental influence of the test setting.

It was studied if modalities of DT and intelligence measures and their interplay affected the relationship between DT and intelligence. However, tests of this moderator were rather inconclusive (i.e., findings were not robust), and future primary studies are clearly needed to shed more light into the issue. As a more general point, it should be noted that common definitions of DT task modality just refer to the content modality of task items and responses, which does not necessarily define the modality of cognitive processes involved in task processing (Benedek and Fink 2019). Especially verbal DT tasks appear to be a quite heterogeneous group, encompassing tasks that require to work creatively with words (e.g., metaphor tasks) or objects (e.g., Alternate Uses Task). Neuroscientific investigations have shown that divergent thinking commonly implicates visual and motor activity, pointing to the involvement of mental simulations (e.g., object manipulations) and visually guided search processes even for verbal material (Benedek et al. 2020; Matheson and Kenett 2020). Future research thus may aim to reconsider established classifications of DT tasks (please note that in the current work also DT task-type did not reveal a differential pattern with respect to the intelligence–DT correlation) with respect to their cognitive demands and eventually concede that few tasks involve only a single modality.

Two aspects of studies’ characteristics moderated the correlations between intelligence and DT: the average age of participants and the gender composition. The links were stronger among older than younger participants and more pronounced in studies composed predominantly by males than females. Although none of these effects was predicted a priori, both seem consistent with the literature.

First, the observed increasing correlations between intelligence and DT with participants’ age are in line with recent studies (e.g., Breit et al. 2020) that found that correlations between different abilities increase with participants’ age. This pattern should be read in light of the long-standing discussion of differentiation-versus-dedifferentiation of cognitive abilities (see, e.g., Hartung et al. 2018). While our findings seem to support the dedifferentiation hypothesis, we acknowledge that meta-analysis is not the best approach to resolve this issue (e.g., ecological fallacy; Viechtbauer 2007). Given that our analyses used the average age of participants and the overlap in age between samples is natural, future studies are needed to more precisely estimate the links between DT and intelligence across different age cohorts. Please note further that for related reasons we even refrained from an analysis of average sample IQ as a moderator. However, the “measurement” of sample age as compared to the measurement of intelligence (see Weiss et al. 2020b) poses fewer challenges in this regard and average sample age can be considered as one of the pertinent sample characteristics used in meta-regression. Hence, while we see problems related to sample age as a moderator and recommend refraining from any straightforward interpretations that generalize from the aggregation level of a meta-analysis to the level of individuals, the found pattern might have heuristic value for the planning of future studies.

The second significant moderation we observed was related to a higher correlation between intelligence and DT in samples composed of males than in samples composed of females. One possible explanation of this pattern refers to the classic “greater male variability hypothesis” (Ellis 1894). Men are characterized by higher variability than women on almost all biological and psychological traits (e.g., Ritchie et al. 2018). Previous studies demonstrated that the males’ variance in intelligence tests is higher than females’ variability (see Wai et al. 2010 or Johnson et al. 2008, for an overview). The same pattern was found in creativity tests, both among children (Karwowski et al. 2016b) and adults (He and Wong 2011). As higher variability strengthens correlations between variables, lower links in predominantly female samples might stem from the restricted variance of intelligence and creativity scores among females. This explanation, however, is tentative and should be directly tested in future studies (i.e., again one should be cautious and not simply generalize from the aggregation level of meta-analysis to the level of individuals).

Interestingly, the observed funnel plot asymmetry was in the opposite direction than one would expect. The observed distribution of coefficients lacked high effect sizes (mostly in the range of highly significant effect sizes) to become symmetrical, indicating that the true relationship between intelligence and DT may be underestimated. The unusual publication bias makes the interpretation difficult. It is possible that changing theoretical assumptions about the relationship between intelligence and DT have influenced what outcomes are desirable. In the 60s of the last century, the prevailing view was that DT and intelligence are virtually unrelated, whereas, at the beginning of the 21st century, the view has shifted to the assumption that DT and intelligence have much more in common than previously thought. Hence, one might conclude that the overall relationship between intelligence and DT is underestimated based on a publication bias that is grounded in a non-significance mechanism, but after correction of the publication bias, the correlation remains moderate (Cohen 1988). In relation to this, it should be further noted that the trim-and-fill method does not work under all conditions (Peters et al. 2007). Hence, given that the Egger-type test revealed a funnel plot asymmetry and the very small increase of the effect size estimate based on the trim-and-fill method, it might also be possible that the corrected estimate is not accurate and caution is needed here. Moreover, beyond a possible publication bias based on non-significance, attention should be paid to the fact that imputed effect sizes on the right sight were all found to be in the region of significant or highly significant correlation coefficients. Peters et al. (2008) suggest that in such situations other factors related to study quality can also cause funnel plot asymmetry. For example, reliability was imputed by means of average reliability across all available estimates, but some studies may have had even lower reliability associated with the used measures. However, without a careful coding and examination of study quality, this issue is not expected to be solved. The found asymmetry could be attributable to a non-significance publication bias, a hidden study quality factor, or a combination of both.

### 5.1. Limitations

Even though this meta-analysis has been prepared carefully in view of the current theories of DT and intelligence research, some limitations must be noted. Several studies did not report sufficient information on DT and intelligence tests, which made the coding procedure difficult. As a result, many intelligence tests were coded with g since there was no other information available. Sometimes it remained unclear which instruction was given in DT tests and, more importantly, how DT outcomes were scored. Consequently, coding might have been more detailed if more information had been provided.

With regard to DT outcomes, a fully differentiated coding was not possible. All subjective scorings were subsumed under the DT outcome *originality*. However, the procedures were not similar in all studies. Splitting up subjective scorings into smaller categories might have provided more insight into the different scoring methods (i.e., top-scoring, snapshot scoring) that are currently being discussed (Reiter-Palmon et al. 2019). It should further be mentioned that explicit instructions to focus on an aspect of creativity (e.g., “be creative”, “be original”) vary in terms of their exact wording (Acar et al. 2020; Runco et al. 2005). In the current work, we did not further distinguish between these subtleties. The main difference between be-creative and be-original instructions, for example, is the wording and while theoretically “creative” does not mean the same thing as “original”, it is far less clear if participants’ understanding of these words reflects the theoretical understanding of creativity researchers. Hence, it is an open question if these nuances in the instructions lead to a different understanding of the task. Indeed, Acar et al. (2020) found different patterns between be-creative and be-original instructions in moderator analyses when effect sizes for mean differences were compared in a meta-analysis on explicit instruction effects. However, how these varying explicit instructions might affect the correlation between DT and intelligence is rather unclear. More intelligent participants might have it easier to adapt to any type of explicit instruction, but then it will depend on the participants’ understanding of the instructions if the intelligence–DT correlation would be affected. Another issue that adds to the complexity of this discussion is instruction-scoring fit (Reiter-Palmon et al. 2019). To conclude, we argue that these types of instructions share a conceptual relation to the most common definitions of creativity, and based on this observation a combined analysis represents a reasonable choice for the context of the current work.

We further did not examine the intelligence–DT correlation as a function of mean IQ because of several methodological problems that could arise from studying it at the level of effect sizes (e.g., Karwowski and Gralewski 2013; Weiss et al. 2020b). The question of a non-linear relationship between creative thinking and intelligence can be better studied by means of complex statistical approaches that are applied in appropriately designed primary studies (e.g., Breit et al. 2020, 2021; Weiss et al. 2020b). Relatedly, other complex interactive effects could be investigated. For example, Harris et al. (2019) proposed that the relationship between creative achievement and intelligence would be moderated by openness to experience. Hence, openness could also be considered as a moderator of the correlation between DT and intelligence. However, measures of openness differ across studies and are unlikely to be on the same scale which in combination with the problems associated with a meta-analytical examination of the threshold hypothesis prevents such a moderator analysis.

As another limitation, we acknowledge that in some instances, moderator analyses relied on a small number of studies. These reported findings should be treated with caution and highlight another goal of meta-analysis, namely the identification of gaps in the empirical study base. Such cells in the moderator analysis design that revealed only few available studies call for further research to strengthen our knowledge on the relationship between DT and intelligence. For example, the proposed interaction between timed vs. untimed testing of DT and intelligence measures was not testable at the level of effect sizes because both measures were never assessed together under untimed conditions. Moreover, with respect to task modality, it was only rarely found that DT assessment involved numerical task content (i.e., only as part of a composite based on measures designed in accordance with the Berlin Structure of Intelligence model; e.g., Preckel et al. 2011), but also pure numerical intelligence measures were only used in very few studies. These observations may pave the way for related future research.

Finally, as mentioned by one anonymous reviewer, under some circumstances (e.g., only categorical moderators are available) it could be a helpful approach to code missing values of moderators as *other* category. However, in most of our meta-regression models mean age and gender ratio were included as moderators and these two variables had the highest proportions of missing values. These two moderators are continuous and such an approach is not applicable without accepting any loss of information in the data (i.e., because of artificially creating categories for these variables). Clearly, complete meta-regression models are desirable to prevent type-I-error associated with separate moderator tests (see Viechtbauer 2007). We agree that this can pose a problem because standard errors in separate moderator tests do not account for the correlational structure among moderators (i.e., moderator confounding) which in turn might yield too liberal statistical inference (i.e., standard errors are underestimated). We argue that our approach to create meta-regressions according to two levels of moderator confounding addresses this issue in a careful way. In addition, our conclusions are quite cautious as it is required for meta-regression which is an approach that has often been criticized (see Viechtbauer 2007). Hence, we are quite confident that for the context of our work moderator analyses are carried out in an appropriate manner.

### 5.2. Recommendations for Future Research

Using the CHC model (Carroll 1997) as a framework for embedding the research efforts about the relationship between intelligence and DT, other subordinate factors must not be overseen. The facet g_r_ (i.e., broad retrieval ability), for instance, reflects the ability to store and later retrieve information fluently through associative processes. Underlying narrow cognitive abilities are (amongst others) ideational fluency, figural flexibility, originality/creativity, and thus abilities that can be accounted to DT as well (McGrew 2009). The first steps investigating the relationship between DT and g_r_ have been made and show substantial associations (e.g., Forthmann et al. 2019; Silvia et al. 2013). Interestingly, recent research suggested that although g_r_ is strongly concerned with verbal fluency, it predicts DT originality at least as strongly as DT fluency (Benedek et al. 2017; Forthmann et al. 2019; Silvia et al. 2013), which may deserve further investigation. Furthermore, g_r_ may benefit from g_c_ and g_f_, and may be a connecting factor when investigating the intelligence–DT relationship, but much more needs to be learned about the inter-relations of the broad cognitive abilities of the CHC model (Carroll 1997). Relatedly, but not discussed in more detail here, the research on executive functions (which has a conceptual overlap with g_r_) and the overlaps with intelligence should be pushed forward. Considering that cognitive flexibility and DT overlap to a certain extent, the impact of working memory and inhibitory control may provide further insight into the underlying cognitive processes. Untangling the theories of intelligence and executive functions might help to locate DT within these constructs. Furthermore, it would be interesting to understand how executive functions and broad cognitive abilities of the CHC model (Carroll 1997) interact and what role g_c_ and g_r_ play within the framework of executive functions.

## 6. Conclusions

The main finding of this study is that the intelligence–DT correlation is very robust, and its size strongly depends on several conditions. This speaks to how cognitive demands during DT and intelligence assessment are affected by these moderators. Hence, if we focus on the concrete choices of creative thinking assessment (i.e., test-like assessments combined with be-creative instructions and scoring of DT originality/creative quality), the intelligence–DT relationship is certainly not as small as in Kim’s (2005) meta-analysis.

## Figures and Tables

**Table 1:** Bivariate correlations between moderators.

Moderator		2	3	4	5	6	7	8	9	10	11	12	13	14
Publication year	1	.36 ^a,^*** (729)	.21 ^a,^*** (604)	−.27 ^a,^*** (476)	**−.60**^a,^*** (247)	.35 ^b,^*** (849)	.66 ^b,^*** (697)	.45 ^b,^*** (808)	**.49**^b,^*** (849)	**.57**^b,^*** (819)	**.73**^b,^*** (712)	.14 ^b,^*** (849)	.27 ^b,^*** (849)	.50 ^b,^*** (849)
Mean age	2		.03 ^a^ (548)	.05 ^a^ (397)	.03 ^a^ (193)	.26 ^b,^*** (729)	.45 ^b,^*** (590)	.36 ^b,^*** (711)	.19 ^b,^*** (729)	**.43**^b,^*** (722)	.23 ^b,^*** (616)	.28 ^b,^*** (729)	.12 ^b,^* (729)	.38 ^b,^*** (729)
Gender ratio	3			−.27 ^a,^*** (285)	−.27 ^a,^*** (209)	.16 ^b,^*** (604)	.36 ^b,^*** (472)	.14 ^b,^** (570)	.27 ^b,^*** (604)	.23 ^b,^*** (577)	.32 ^b,^*** (494)	.13 ^b,^* (604)	.15 ^b,^** (604)	.31 ^b,^*** (604)
DT time-on-task	4				.01 ^a^ (227)	.07 ^b^ (476)	.35 ^b,^*** (432)	.04 ^b^ (476)	.33 ^b,^*** (476)	NA	**.58**^b,^*** (444)	.31 ^b,^*** (476)	.10 ^b^ (476)	.48 ^b,^*** (476)
Intelligence time-on-task	5					.39 ^b,^*** (247)	**.45**^b,^*** (237)	.11 ^b^ (246)	**.40**^b,^*** (247)	.15 ^b^ (247)	NA	.13 ^b^ (247)	.24 ^b,^** (247)	.53 ^b,^*** (247)
Intelligence facet	6						.35 ^c,^*** (697)	.20 ^c,^*** (808)	.25 ^c,^*** (849)	.27 ^c,^*** (819)	.19 ^c,^*** (712)	.16 ^c,^*** (849)	**.55**^c,^*** (849)	.25 ^c,^*** (849)
DT instruction	7							NA	.41 ^c,^*** (697)	.47 ^c,^*** (668)	.42 ^c,^*** (575)	.45 ^c,^*** (697)	.25 ^c,^*** (697)	.34 ^c,^*** (697)
Other aspects of DT instruction	8								.18 ^c,^*** (808)	.58 ^c,^*** (808)	.28 ^c,^*** (700)	.17 ^c,^*** (808)	.14 ^c,^*** (808)	.41 ^c,^*** (808)
DT scoring	9									.31 ^c,^*** (819)	.33 ^c,^*** (712)	.19 ^c,^*** (849)	.15 ^c,^*** (849)	.34 ^c,^*** (849)
DT time condition	10										.40 ^c,^*** (711)	.26 ^c,^*** (819)	.20 ^c,^*** (819)	.46 ^c,^*** (819)
Intelligence time condition	11											.30 ^c,^*** (712)	.27 ^c,^*** (712)	.41 ^c,^*** (712)
DT modality	12												.20 ^c,^*** (849)	.62 ^c,^*** (849)
Intelligence modality	13													.21 ^c,^*** (849)
DT task-type	14													

Number of coefficients *k* is provided in parentheses. Correlation coefficients ≥.40 are highlighted in bold (please note that correlations were rounded to two decimals and some values depicted as .40 are not in bold font because they resulted from rounding). ^a^ Pearson correlation coefficient. ^b^ coefficient η. ^c^ Cramer’s *V*. * *p* < .05. ** *p* < .01. *** *p* < .001.

**Table 2:** Effect sizes as a function of intelligence facet.

		95%-CI					
Effects	Estimate	LB	UB	*p*	*k*	*m*	*N*
g	.28	.23	.33	<.001	245	52	11,145
g_c_	.28	.22	.33	<.001	137	28	4600
g_f_	.23	.18	.28	<.001	467	67	26,765

LB = lower bound; UB = upper bound; *k* = number of coefficients; *m* = number of studies; *N* = total sample size.

**Table 3:** Effect sizes as a function of DT instructions.

		95%-CI					
Effects	Estimate	LB	UB	*p*	*k*	*m*	*N*
Be-fluent	.25	.21	.30	<.001	346	59	17,255
Be-original/creative	.29	.22	.35	<.001	166	14	1930
Hybrid-fluent-flexible	.24	.13	.34	<.001	32	8	2451
Hybrid-fluent-original	.21	.13	.29	<.001	73	4	1060
Hybrid-flexible-original	.23	.03	.42	.026	19	2	458
Hybrid-fluent-flexible-original	.23	.03	.41	.022	21	3	570
Mixed	.26	.14	.37	<.001	40	5	1603

LB = lower bound; UB = upper bound; *k* = number of coefficients; *m* = number of studies; *N* = total sample size.

**Table 4:** Effect sizes as a function of other aspects of DT instructions.

		95%-CI					
Effects	Estimate	LB	UB	*p*	*k*	*m*	*N*
Game-like instructions	.07	−.02	.16	.112	137	10	1998
Test-like instructions	.27	.22	.31	<.001	647	100	30,682
Strategy instructions	.33	.22	.43	< 001	24	3	285

LB = lower bound; UB = upper bound; *k* = number of coefficients; *m* = number of studies; *N* = total sample size.

**Table 5:** Effect sizes as a function of DT scoring.

		95%-CI					
Effects	Estimate	LB	UB	*p*	*k*	*m*	*N*
Fluency	.20	.15	.25	<.001	564	76	24,927
Originality/creative quality	.31	.24	.37	<.001	190	26	4977
Composite score	.37	.29	.45	<.001	73	27	5226

LB = lower bound; UB = upper bound; *k* = number of coefficients; *m* = number of studies; *N* = total sample size.

**Table 6:** Effect sizes as a function of DT instruction-scoring fit (Reiter-Palmon et al. 2019).

		95%-CI					
Effects	Estimate	LB	UB	*P*	*k*	*m*	*N*
Be-fluent—Fluency	.22	.15	.28	<.001	262	41	10,870
Be-fluent—Originality/creative quality	.26	.17	.34	=.001	44	13	4818
Be-original/creative—Fluency	.17	.07	.27	<.001	31	9	1349
Be- original/creative—Originality/creative quality	.34	.27	.43	<.001	132	12	1790

LB = lower bound; UB = upper bound; *k* = number of coefficients; *m* = number of studies; *N* = total sample size.

**Table 7:** Effect sizes as a function of DT modality and intelligence modality of the measures.

			95%-CI					
DT Modality	Intelligence Modality	Estimate	LB	UB	*p*	*k*	*m*	*N*
Verbal	Verbal	.25	.20	.30	<.001	242	45	12,381
Figural	Verbal	.14	.07	.22	<.001	53	15	4425
Several	Verbal	.28	.14	.42	<.001	19	8	1419
Verbal	Numerical	.23	.16	.31	<.001	51	8	2207
Figural	Numerical	.08	−.10	.25	=.397	6	4	1611
Several	Numerical	.24	−.21	.60	=.295	1	1	239
Verbal	Figural	.22	.17	.28	<.001	182	31	5548
Figural	Figural	.16	.08	.25	<.001	48	13	2897
Several	Figural	.28	.14	.40	<.001	15	12	3430
Verbal	Several	.30	.25	.35	<.001	121	37	14,426
Figural	Several	.19	.11	.27	<.001	59	14	2595
Several	Several	.33	.25	.41	<.001	52	17	4213

LB = lower bound; UB = upper bound; *k* = number of coefficients; *m* = number of studies; *N* = total sample size.

**Table 8:** Effect sizes as a function of DT task-type.

		95%-CI					
DT Task-Type	Estimate	LB	UB	*p*	*k*	*m*	*N*
AUT	.25	.19	.31	<.001	287	45	7926
Consequences	.37	.23	.49	<.001	13	8	9290
Instances	.36	.27	.45	<.001	32	7	988
Line meanings	.27	.16	.37	<.001	29	6	693
Other	.30	.21	.39	<.001	42	8	1361
Pattern meanings	.20	.11	.29	<.001	43	6	624
Several	.31	.24	.37	<.001	116	29	7068
Similarities	.34	.24	.44	<.001	27	5	573
Test battery	.18	.11	.25	<.001	260	37	11,757

LB = lower bound; UB = upper bound; *k* = number of coefficients; *m* = number of studies; *N* = total sample size.

**Table 9:** Robustness checks.

	Correlation between Confounding Moderators Must Be > .40			Correlation between Confounding Moderators Must Be > .20		
Moderator Effect	Number of Confounding Moderators (*k*/Number of Model Coefficients)	Rule of Thumb Okay?	Effect Robust?	Number of Confounding Moderators (*k*/Number of Model Coefficients)	Rule of Thumb Okay?	Effect Robust?
Main effect—Other DT instruction aspects	3 (808/13)	Yes	Yes	6 (604/17)	Yes	Fairly
Main effect—DT scoring	2 (695/10)	Yes	Fairly	7 (376/23)	Yes	Fairly
**Instruction-Scoring fit**	**5 (324/10)**	**Yes**	**Yes**	**9 (246/24)**	**Yes**	**Yes**
Main effect—DT modality ^a^	3 (697/22)	Yes	Fairly	7 (492/26)	Yes	No
Main effect—DT task-type	7 (700/16)	Yes	No	9 (451/22)	Yes	No
**Main effect**—**mean age**	**2 (584/9)**	**Yes**	**Yes**	**7 (492/23)**	**Yes**	**Yes**
**Main effect**—**gender ratio ^b^**	**-**	**-**	**-**	**6 (377/23)**	**Yes**	**Yes**

Rule of thumb = k > 100 + number of model coefficients (Green 1991). ^a^ The observation that figural DT correlated less strong with intelligence as compared to verbal DT was based on a model including both modality main effects and the robustness checks here were also based on such a model. ^b^ No other moderator correlated with gender ratio >.40 (see Table 1). Bold lines refer to results that were fully robust.

## Data Availability

The data can be accessed at https://osf.io/s4hx5.
